# A case report of leishmaniosis with primary oral manifestation in a cat

**DOI:** 10.3389/fvets.2022.1059803

**Published:** 2022-11-29

**Authors:** Lisa A. Mestrinho, Joana Travancinha, Cristina Sobral

**Affiliations:** ^1^CIISA—Centre for Interdisciplinary Research in Animal Health, Faculty of Veterinary Medicine, University of Lisbon, Lisbon, Portugal; ^2^Laboratório Associado para Ciência Animal e Veterinária (AL4AnimalS), Lisbon, Portugal; ^3^Vetalmada, Veterinary Clinic, Almada, Portugal

**Keywords:** *Leishmaniosis*, oral lesions, focal stomatitis, vector born disease, feline

## Abstract

A case of leishmaniosis with primary oral manifestations was reported in a 10-year-old neutered domestic shorthair cat. The primary lesion was a maxillary nodular lesion, painful with spontaneous bleeding associated with advanced periodontal disease, which did not resolve with tooth extraction or periodontal treatment. Biopsy revealed chronic neutrophilic and macrophagic infections and amastigote forms of *Leishmania* sp and molecular tests were able to identify *Leishmania infantum*. Oral signs resolved after the initiation of etiologic treatment with allopurinol. Distinguishing oral signs of leishmaniosis from other oral inflammatory diseases is important, especially in endemic areas, and co-infections must be considered with any oral manifestations of this disease.

## Introduction

*Leishmania infantum* infection in cats is less frequent due to the natural resistance of this species to the parasite, and dogs are the main reservoir of this protozoan. Clinical manifestations are mainly dermatological or visceral, although oral lesions such as gingivitis, stomatitis, and periodontitis have also been reported (1). To the best of our knowledge, there are no detailed cases of the primary oral manifestations of feline leishmaniosis. This case report describes the clinical, radiographic, and histological findings of a cat presenting with primary oral signs of leishmaniosis.

## Case description

**Figure 1:**
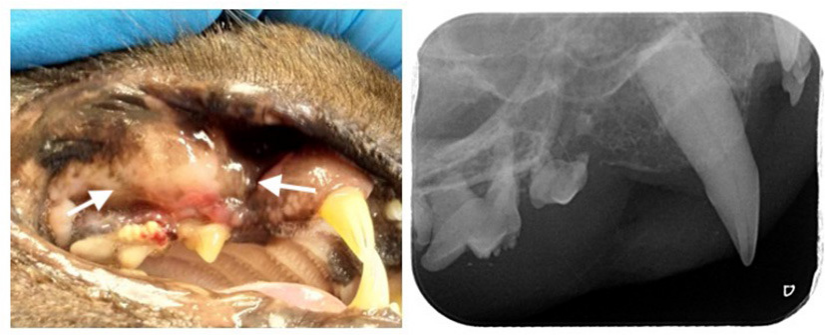
Leishmaniosis nodular ulcero-hemorrhagic lesion (arrows) identified in the right maxilla in a 10-year-old cat (left), intra-oral radiography of the right maxillary premolars and molars where it is possible to identify tooth resorption and vertical bone loss associated with the lesion (right).

A 10-year-old, male neutered, domestic shorthair cat (weight: 5.2 kg, body condition score of 4 out of 9) presented with a complaint of halitosis during consultation. Weight change was not noted, although the animal showed occasional signs of discomfort and difficulty in eating for some weeks. The animal was a rescued cat with previous outdoor access. Presently he was living an indoor with no other animals sharing the environment. Ecto- and endoparasite prophylaxis was up to date. At time of adoption immunological tests for feline immunodeficiency virus (FIV) and feline leukemia virus (FeLV) were performed and scored negative. Clinical examination revealed a periodontal disease more severe on the right side and a right maxillary local stomatitis with nodular and ulcerative appearance (Figure 1). On inspection, the nodular lesion was slightly painful with spontaneous bleeding. Bilateral mandibular lymph node enlargement was also observed. No other anomalies were observed.

Preoperative blood analysis included a complete blood count (CBC), biochemistry, and total T4 levels. Leukopenia [4.93 × 10^3^/μl; Reference range (RR) 5.5–19.5], elevated total protein (10.06 g/dl; RR 6.0–7.9) and globulins (7.76 g/dl; RR 2.6–5.1), hypoalbuminemia (2.3 g/dl; RR 2.8–3.9), mild hyperbilirubinemia (0.12 mg/dl; RR 0.0–0.1), with no other blood abnormalities were found. Surgery was scheduled.

The cat was anesthetized using a combination of dexmedetomidine (Dexdomitor, Ecuphar, 2 μg/kg), ketamine (Ketomidor, Richter Pharma, 2.5 mg/kg), and methadone (Senfortam, Ecuphar, 0.2 mg/kg). Induction was performed using propofol (Propovet, Ecuphar, 1 mg/kg) and maintained with isoflurane after intubation. Intravenous support was provided using a cephalic vein catheter with Ringer lactate at a dosage of 5 ml/kg during the operatory period. Bilateral mandibular and maxillary nerve blocks were performed by administering 2% lidocaine at each location.

A detailed oral examination during anesthesia and full-mouth radiography were performed. Periodontal pocket depth at the level of the nodular ulcero-hemorrhagic lesion (third right maxillary premolar tooth) was 7 mm, and the second right premolar tooth was absent. Advanced periodontitis was diagnosed in all the right maxillary premolars and molars, both mandibular molars and left third mandibular premolar, and third right mandibular incisors. Moderate periodontitis in the left maxillary fourth premolar and early periodontitis in all canine teeth. Tooth resorption was also identified in six teeth, including the right maxillary third premolar. At the level of the nodular ulcero-hemorrhagic lesion, the third and fourth right maxillary premolars were affected by advanced periodontitis, and radiographic signs of vertical bone loss were observed (Figure 1). Treatment included the extraction of teeth with tooth resorption and advanced attachment loss. Standard surgical extraction techniques were performed and the gingival flaps were routinely closed using a simple interrupted pattern with absorbable sutures. Incisional biopsies of the maxillary lesions were performed for further histopathological assessment.

Post-operatory medication included meloxicam (Loxicom, Norbrook, 1 mg/kg) and buprenorphine (Buprenodale, Decra, 2 μg/kg) every 12 h for 5 days, clindamycin (Clindaseptin, Vetoquinol) 5.5 mg/kg every 12 h for 10 days, chlorhexidine 0.12% (Stomodine F, ICF) topical every 12 h for 15 days.

**Figure 2:**
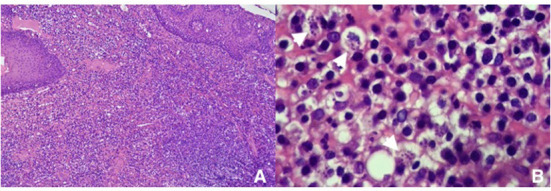
Histopathology photomicrograph of the biopsy, showing amastigote figures and neutrophilic and macrophagic infiltration **(A)** 100× and **(B)** 400×.

Histopathological studies revealed ulcerous-hemorrhagic gingivitis associated with bacterial infection and an abundance of amastigote forms of *Leishmania*. Inflammatory cells were mainly neutrophils and macrophages, with a lower proportion of lymphocytes and plasmacytes (Figure 2).

Two weeks after surgery, the owners reported a normal appetite and no halitosis. Weight had also improved (5.35 kg). On oral inspection, a nodular ulcero-hemorrhagic lesion was still observed, associated with persistent swelling and inflammation in the right maxilla.

Further analysis was proposed by performing an initial enzyme immune assay (Leiscan, Esteve Pharma). After being confirmed positivity, an antibody titer of 1:640 was determined byimmunofluorescence antibody test (IFAT). Additionally, quantitative PCR (EXOone Leishmania spp. Onemix, Exopol, Spain) was requested in blood and gingival tissues, which confirmed *L. infantum*. Monoclonal gammopathy was identified on the proteinogram. A definitive diagnosis of leishmaniosis was made, and a treatment plan was accepted by the owner, which included allopurinol at a total dosage of 100 mg once daily. Routine blood analysis included CBC, biochemical analysis, urinalysis, urine protein into creatinine, and proteinogram every 15 days for the first month, followed by every 3 months for a year, and every 6 months for 2 years.

Mild diarrhea, polyuria, and polydipsia were noted initially but reversed after 2 and 4 weeks. Table 1 summarizes proteinogram levels at day 0, 90, and 180. The oral lesion completely resolved after 1 month. Proteinograms showed improvement after 15 days of allopurinol treatment, with decreasing globulin levels 8 months after treatment. Leukopenia remained unchanged. The patient remains under monitoring 2 years after diagnosis. Chronic kidney disease was diagnosed 1 year after diagnosis.

**Table 1:** Proteinogram values registered at day 0, 90 and 180.

	**Day 0**	**Day 90**	**Day 180**	**Reference range (g/dl)**
Total proteins	10	8.5	7.8	6.6–8.4
Albumin	2.53	3.41	3.41	2.94–4.48
Alfa 1	0.11	0.17	0.09	0.07–0.32
Alfa 2	0.79	0.94	0.78	0.54–1.35
Beta 1	0.72	0.48	0.47	0.25–0.88
Beta 2	0.4	0.42	0.37	0.23–0.87
Gama	5.88	3.09	2.66	0.35–1.87
Ratio albumin/globulin	0.34	0.67	0.78	0.79–1.52

## Discussion

Natural infection with *L. infantum* is less frequently described in cats because of its natural resistance to this parasite (2). Clinical signs are more discrete than those in dogs, ranging from systemic to local skin lesions (2). Most lesions are cutaneous, specifically pinna and periocular, in the head area (3). Gum lesions have also been described as clinical signs of leishmaniosis (1). Some authors describe lesions as chronic gingivostomatitis (CGS) (1, 4, 5) and others as mucocutaneous nodular lesions (6–8). In this case, the oral mucosal lesion had a nodular appearance extending from the gingiva to the buccal mucosa (stomatitis), and was unilateral, ulcerative, hemorrhagic, and painful on inspection, in contrast to the bilateral generalized pattern found in CGS. Additionally, periodontal disease and tooth resorption are closely associated with nodular stomatitis lesions, as confirmed by increased pocket depth and radiographic evidence of vertical bone loss and external tooth resorption. Bone loss can be a sign of osteomyelitis, which is frequently observed in periodontal diseases.

Regardless of the identification of periodontal disease and tooth resorption, caudal stomatitis is a lesion pattern that distinguishes CGS in cats from other oral conditions (9); however, this finding was not observed in this case. Considering the localized pattern of the lesion observed here and those observed in previous literature (1, 6), localized nodular lesions are probably primary lesions secondary to *Leishmania* infection. CGS is a multifactorial disease in which chronic viral antigenic stimulation seems to be a strong contributor to antigenic stimulation that leads to specific histopathological features (9–12). Contrary to what is normally observed in the histopathology of CGS biopsies, a lymphoplasmacytic infiltrate with frequent Mott bodies (9, 11), neutrophilic and macrophage infiltration, and a lower proportion of lymphocytes and plasmacytes were observed in this case. The presence of Mott bodies is associated with viral infections. In contrast to the antigenic stimulation of leishmaniosis, which is a protozoan, the inflammatory reaction is mainly related to macrophage infiltration and neutrophilic due to secondary bacterial infection.

Other diseases can predispose cats to develop leishmaniosis (2, 13–15). Some authors have identified a strong association between FIV positivity in cats and *Leishmania* infection (14–17). Indeed, in the presence of co-infection (FIV-*Leishmania*), oral lesions are the most frequent finding (15). Interestingly, extensive oral lesions were found in a cat immunosuppressed after corticosteroid therapy and was infected with *Leishmania* (7). In our case report, common screened retroviral diseases (FIV and FeLV) were negative, and other co-infections were not detected clinically; however, the advanced age of the animal might have increased the risk of infection if exposed to the vector. Other factors include the animal's lifestyle, previous outdoor access in a periurban endemic area, increased the chance to vector exposure.

It is possible that periodontal disease and tooth resorption could be strong contributors to the oral manifestation of leishmaniosis, as both these diseases can cause pain. Regardless, even after periodontal treatment and tooth extractions, the suspected lesion did not resolve despite the overall post-treatment improvement on the contralateral side of the oral cavity. Therefore, not all signs can be related to leishmaniosis, since many cats have comorbidities (13–17). Primary localized lesions can become more severe and extensive in the presence of a comorbidity, which, in turn, contributes to generalized immunosuppression and decreases the primary resistance of cats to this parasite. It may be interesting to evaluate in future case series if leishmaniosis oral lesions are primary nodular or generalized, and try to distinguish if a more generalized pattern is linked to immunosuppression.

Finally, hypoalbuminemia and monoclonal hypergammaglobulinemia were consistent findings that changed throughout the course of treatment. This finding is indicative of systemic alterations. Furthermore, 1 year after the initial diagnosis and beginning of treatment, the cat developed chronic kidney failure. This might be related to the evolution of visceral leishmaniosis or other events, such as immune complex deposition, pharmacologic effects related to allopurinol treatment, or sclerotic kidney disease. In a previous case series, *L. infantum* infection was associated with kidney disease (17).

In conclusion, in the presence of nodular stomatitis lesions in endemic areas, leishmaniosis must be considered, and it is mandatory to investigate any possible comorbidities contributing to the clinical manifestation of this disease. Local treatment of these lesions is insufficient and therefore etiological treatment is necessary.

## Data availability statement

The raw data supporting the conclusions of this article will be made available by the authors, without undue reservation.

## Ethics statement

Ethical review and approval was not required for the animal study. Written informed consent was obtained from the owners for the participation of their animals in this study.

## Author contributions

All authors listed have made a substantial, direct, and intellectual contribution to the work and approved it for publication.

## Funding

This work was funded by FCT—Fundação para a Ciência e Tecnologia, grant UIDB/00276/2020, from CIISA—Centro de Investigação Interdisciplinar de Sanidade Animal, Faculdade de Medicina Veterinária, Universidade de Lisboa, and LA/P/0059/2020 - AL4AnimalS, Portugal.

## Conflict of interest

The authors declare that the research was conducted in the absence of any commercial or financial relationships that could be construed as a potential conflict of interest.

## Publisher's note

All claims expressed in this article are solely those of the authors and do not necessarily represent those of their affiliated organizations, or those of the publisher, the editors and the reviewers. Any product that may be evaluated in this article, or claim that may be made by its manufacturer, is not guaranteed or endorsed by the publisher.
